# Variation in the Bioactive Compound Content at Three Ripening Stages of Strawberry Fruit

**DOI:** 10.3390/molecules190710370

**Published:** 2014-07-17

**Authors:** Sandra Voća, Jana Šic Žlabur, Nadica Dobričević, Lidija Jakobek, Marijan Šeruga, Ante Galić, Stjepan Pliestić

**Affiliations:** 1 Department of Agricultural Technology, Storage and Transport, Faculty of Agriculture, University of Zagreb, Svetošimunska cesta 25, HR-10000 Zagreb, Croatia; E-Mails: svoca@agr.hr (S.V.); ndobricevic@agr.hr (N.D.); agalic@agr.hr (A.G.); spliestic@agr.hr (S.P.); 2 Department of Applied Chemistry and Ecology, Faculty of Food Technology, J. J. Strossmayer, University of Osijek, Kuhačeva 18, HR-31000 Osijek, Croatia; E-Mails: lidija.jakobek@ptfos.hr (L.J.); marijan.seruga@ptfos.hr (M.S.)

**Keywords:** *Fragaria* x *ananassa* Duch., harvest period, anthocyanin content, phenolic acids, flavonoids

## Abstract

During the harvest season of two consecutive years, five strawberry cultivars (‘Arosa’, ‘Elsanta’, ‘Marmolada’, ‘Miss’ and ‘Raurica’), grown in the continental part of the Republic of Croatia, were examined. Strawberry fruits quality was evaluated by individual phenol compounds, individual anthocyanins and fruit color. Fruits were harvested in three different periods. Analyzed strawberry cultivars show very good average values of the studied phenolic acids and flavonoids with predominant caffeic acid and epicatechin content in all researched strawberry cultivars. Considering the content of individual anthocyanins, pelargonidin 3-glucoside is predominant in strawberry extract followed by cyanidin-3-glucoside and pelargonidin 3-rutinoside. The correlation between individual anthocyanin content and chromaticity parameters was detected in all strawberry cultivars, additionally correlation coefficients and statistical significance were much lower. The results show a positive association between cultivar and harvest time on strawberry pulp color, with each of the color variables, *a*, *b*, *a/b* ratio, *C*, *L* and *h°* values.

## 1. Introduction

Strawberry fruit (*Fragaria* x *ananassa* Duch.) is rich source of vitamins and beneficial dietary compounds. Worldwide, strawberry is one of the most popular fruits for human consumption, appreciated for its unique flavour. In addition to the usual nutrients, such as vitamins and minerals, strawberries are rich in anthocyanins, flavonoids and phenolic acids [1,2]. Besides sweetness, acidity and flavour, the development of red or scarlet color is one of the most important characteristics affecting strawberry fruit quality, as well as contributing to a consumer’s initial impression [3]. The ripening process of strawberry fruits not only strongly affects the favourable nutrition composition of fruits but also contributes to the creation of the characteristic fruit taste. To achieve the maximum quality in terms of fruit flavor and color, strawberry fruits must be harvested at stage of full maturity. The final ripening stage of the fruit comprises a series of biochemical and physiological processes which *inter alia* include: modification of cell walls, the conversion of starch into sugars, alterations in pigment biosynthesis, accumulation of flavour and aromatic volatiles as well as heightened level of polyphenols and other antioxidant compounds [4,5]. Harvest dynamics of strawberry fruits have a very important role in terms of fruit quality and yield quantity. Strawberry genotype as well as harvest period and other factors such as: selection of mulch, air temperature and humidity during the maturation period, irrigation and plant protection have strong influences on harvest dynamics. Maturation dynamics and harvest period are the main factors influencing the significant differences between cultivation years [6].

The characteristic strawberry fruit color results from the pigment compounds anthocyanins. Different studies have shown that strawberries have a simple anthocyanin profile, with pelargonidin 3-glucoside (PG) as predominant pigment, followed by cyanidin 3-glucoside (CG) [3,7]. Pelargonidin 3-glucoside (PG), the major anthocyanin compound found in strawberries is most responsible for the bright red color of fresh strawberries [3]. Attractive color is a very important sensory characteristic for the fresh consumption of fruits as well as it for the processed strawberry food products such as juice, jam, dehydrated fruits, *etc.*, Anthocyanins show a significant nutritional value directly associated with a number of benefits for human health ranging from antioxidant potential, anticancer activity, anti-inflammatory and anti-angiogenic properties [8,9,10,11,12,13,14]. Strawberry genotype strongly correlates with the anthocyanin profile present in fruits [15]. Various studies have shown the significance of strawberry germplasm on the nutritional and sensory parameters of the fruits [16]. The aim of this study was to investigate the changes in the quantity of anthocyanins and phenols content during the harvest season, and how the composition of anthocyanins, mainly pelargonidin 3-glucoside, pelargonidin 3-rutinoside and cyanidin 3-glucoside affects fruit color.

## 2. Results

### 2.1. The Individual Anthocyanin Composition of Selected Strawberry Varieties

Results of analyzed individual anthocyanins in different strawberry cultivars are shown in Table 1. The Pg 3-gluc was identified as the major anthocyanin in all analyzed cultivars. The highest value of Pg 3-gluc (432.28 mg kg^−1^) was measured in variety ‘Marmolada’ in the third harvest period second research year while the lowest value (107.94 mg kg^−1^) was in the variety ‘Raurica’ in the third harvest period of the first research year. The lowest values for Pg 3-gluc content were noted in both research years for cultivars ‘Elsanta’, ‘Miss’ and ‘Raurica’. The Cy 3-gluc concentration ranged from 35.90 to 88.41 mg kg^−1 ^ in the first research year and from 35.31 to 87.13 mg kg^−1 ^ in the second year. The highest value of the Cy 3-gluc was noted in the ‘Arosa’, ‘Raurica’ and ‘Elsanta’ cultivars, while the lowest values were noted in ‘Marmolada’ and ‘Miss’ cultivars. The concentration of Pg 3-gluc were also different depending on the studied cultivars. Concentrations of Pg 3-rut were more even. Lower values were noted in cultivars ‘Raurica’ (23.65–64.32 mg kg^−1^) and ‘Marmolada’ (34.37–61.24 mg kg^−1^) in the first year. From all analyzed individual anthocyanins high statistically significant differences between strawberry variety and harvest period in both research years were determined.

**Table 1:** Content of individual anthocyanins (mg kg^−1^) in selected strawberry varieties harvested in three harvest periods during two research years.

First research year				
Strawberry cultivar	Harvest period	Cyanidin 3-glucoside **	Pelargonidin 3-glucoside **	Pelargonidin 3-rutinoside **
Arosa	1	81.23 ^b^	321.54 ^a^	55.21 ^b^
	2	82.87 ^a^	317.18 ^b^	50.92 ^b^
	3	82.66 ^a^	309.08 ^b^	85.33 ^a^
Elsanta	1	72.32 ^b^	234.32 ^b^	46.79 ^c^
	2	78.02 ^b^	222.25 ^b^	57.65 ^b^
	3	86.29 ^a^	254.98 ^a^	67.17 ^a^
Marmolada	1	45.21 ^b^	325.11 ^a^	61.24 ^a^
	2	48.10 ^b^	263.39 ^b^	50.11 ^b^
	3	68.42 ^a^	336.49 ^a^	34.37 ^c^
Miss	1	66.94 ^a^	194.84 ^c^	81.94 ^a^
	2	35.90 ^b^	241.82 ^b^	68.21 ^b^
	3	40.42 ^b^	255.78 ^a^	59.15 ^b^
Raurica	1	85.08 ^a^	220.61 ^a^	64.32 ^a^
	2	75.42 ^b^	177.25 ^b^	35.81 ^b^
	3	88.41 ^a^	107.94 ^b^	23.65 ^b^
**Second research year**				
Arosa	1	78.73 ^b^	205.18 ^c^	51.67 ^a^
	2	76.28 ^b^	318.37 ^b^	37.97 ^b^
	3	87.13 ^a^	385.66 ^a^	40.63 ^b^
Elsanta	1	70.32 ^a^	245.21 ^a^	41.23 ^b^
	2	64.24 ^b^	235.83 ^b^	48.87 ^b^
	3	68.08 ^a^	240.75 ^a^	58.10 ^a^
Marmolada	1	44.85 ^b^	420.17 ^b^	64.90 ^a^
	2	46.30 ^b^	260.51 ^c^	42.28 ^b^
	3	56.50 ^a^	432.28 ^a^	39.76 ^b^
Miss	1	50.81 ^a^	201.82 ^b^	55.12 ^a^
	2	39.06 ^b^	259.44 ^a^	44.86 ^b^
	3	35.31 ^b^	252.10 ^a^	56.78 ^a^
Raurica	1	69.30 ^a^	245.14 ^b^	41.93 ^a^
	2	57.89 ^b^	227.21 ^c^	39.59 ^a^
	3	58.52 ^b^	278.49 ^a^	17.12 ^b^

Results with different letters are significantly different (*p* < 0.05); ** *p* < 0.01.

### 2.2. Phenolic Compounds Determined in Selected Strawberry Varieties

The content of select three phenolic acids (caffeic, chlorogenic and ellagic acid) and four flavonoids (epicatehin, catehin, procyanidin B2 and rutin) are shown in Table 2. High statistically significant differences were determined in five strawberry cultivars for analyzed phenolic compounds in both research years. Among the analyzed phenolic acids in strawberry fruits, caffeic acid is predominant. Caffeic acid content is the highest in all analyzed strawberry varieties compared with other determined phenolic acids. According to the presence, chlorogenic acid and ellagic acid follow with slight modifications in the variety ‘Marmolada’ in the second and third harvest period (Table 2). Variety ‘Arosa’ through all three harvest periods in both research years had the highest average value of caffeic acid (65.84 mg kg^−1 ^ in the first year; 65.26 mg kg^−1 ^ in the second research year). The amount of chlorogenic acid was statistically significantly different considering the strawberry variety and harvest period in range from 6.17 to 81.22 mg kg^−1 ^ in the first year, and 6.80 to 97.43 mg kg^−1 ^ in the second research year. The minimum values of chlorogenic acid were determined in both research years in the variety ‘Marmolada’ in the second and third harvest period. The highest value of chlorogenic acid was determined in the varietiy ‘Arosa’ in the first harvest period in both research years. Ellagic acid amounts were uniform in both years. Variety ‘Miss’ had the highest value of extracted ellagic acid in both years in the first harvest period. The amount of epicatechin was 20.60 to 93.17 mg kg^−1^ in the first and 12.82 to 84.45 mg kg^−1^ in the second research year. The highest values were observed in fruits of strawberry variety ‘Miss’ in the third harvest period in both years. Average values of catechin were uniform in both years, regardless of the harvest period. The amount of procyanidin B2 was the highest in ‘Arosa’ in the second harvest period in both research years. Quantity of rutin ranged from 4.07 mg kg^−1^ to 85.12 mg kg^−1^ in the first and from 4.83 mg kg^−1^ to 96.74 mg kg^−1^ in the second research year. The highest value of rutin was recorded in variety ‘Arosa’ in the first harvest period in both years. The quantity of rutin was uneven, depending on the variety and harvest period. 

**Table 2:** Phenolic compounds content (mg kg^−1^) in samples of selected strawberry varieties harvested in three harvest periods during two research years.

First Research Year																	
Phenolic Acid									Flavonoids								
Strawberry Cultivar		Harvest Period	Caffeic Acid		Chlorogenic Acid		Ellagic Acid		Epicatechin		Catechin		Procyanidin B_2_		Rutin		
		**	**		**		**		**		**		**		**		
Arosa		1	85.22 ^a^		81.22 ^a^		24.17 ^a^		42.12 ^b^		72.14 ^a^		72.01 ^b^		85.12 ^a^		
		2	57.91 ^b^		32.38 ^b^		24.26 ^a^		44.98 ^b^		36.11 ^c^		84.07 ^a^		30.97 ^b^		
		3	54.39 ^b^		34.93 ^b^		22.43 ^b^		83.89 ^a^		65.74 ^b^		51.74 ^c^		35.25 ^b^		
Elsanta		1	29.62 ^b^		28.18 ^a^		23.53 ^a^		64.79 ^b^		53.19 ^a^		28.42 ^a^		40.01 ^a^		
		2	42.85 ^a^		18.99 ^b^		21.46 ^ab^		82.32 ^a^		40.60 ^b^		21.20 ^b^		4.07 ^c^		
		3	22.44 ^c^		30.52 ^a^		20.99 ^ab^		83.26 ^a^		52.59 ^a^		28.73 ^a^		13.91 ^b^		
Marmolada		1	41.22 ^a^		25.12 ^a^		22.61 ^a^		52.11 ^a^		68.29 ^a^		33.22 ^a^		39.11 ^b^		
		2	13.11 ^c^		6.17 ^b^		21.51 ^ab^		49.54 ^a^		26.46 ^c^		30.74 ^a^		27.65 ^c^		
		3	24.07 ^b^		8.48 ^b^		22.54 ^a^		20.60 ^c^		43.52 ^b^		24.18 ^b^		47.24 ^a^		
Miss		1	70.57 ^a^		40.32 ^b^		25.74 ^a^		58.22 ^b^		72.82 ^a^		23.43 ^b^		74.08 ^a^		
		2	31.51 ^b^		50.30 ^a^		22.10 ^ab^		51.15 ^b^		51.27 ^b^		Trace ^c^		6.01 ^c^		
		3	17.03 ^c^		12.67 ^c^		20.73 ^ab^		93.17 ^a^		30.85 ^c^		52.45 ^a^		21.08 ^b^		
Raurica		1	24.64 ^a^		15.87 ^b^		23.22 ^a^		47.15 ^a^		67.60 ^a^		68.99 ^a^		64.32 ^a^		
		2	12.86 ^b^		27.10 ^a^		21.90 ^ab^		45.43 ^ab^		33.36 ^b^		17.84 ^c^		16.79 ^c^		
		3	14.23 ^b^		20.37 ^ab^		21.33 ^ab^		43.65 ^b^		34.99 ^b^		43.27 ^b^		32.50 ^b^		
**Second research year**																	
Arosa		1	92.73 ^a^		97.43 ^a^		22.13 ^ab^		41.11 ^b^		85.30 ^a^		75.78 ^b^		96.74 ^a^		
		2	34.73 ^c^		14.67 ^c^		21.70 ^ab^		30.96 ^c^		37.17 ^c^		89.52 ^a^		23.74 ^c^		
		3	68.33 ^b^		40.36 ^b^		23.84 ^a^		63.82 ^a^		62.52 ^b^		58.61 ^c^		55.92 ^b^		
Elsanta		1	30.16 ^a^		31.16 ^a^		23.18 ^a^		61.82 ^a^		50.29 ^a^		31.01 ^a^		35.22 ^a^		
		2	26.69 ^b^		23.30 ^ab^		21.21 ^ab^		51.99 ^a^		43.47 ^ab^		24.65 ^b^		12.96 ^b^		
		3	16.65 ^c^		14.35 ^b^		20.71 ^ab^		23.73 ^b^		37.02 ^b^		30.02 ^a^		10.46 ^b^		
Marmolada		1	43.99 ^a^		33.40 ^a^		23.47 ^a^		47.69 ^a^		66.77 ^a^		29.01 ^a^		41.81 ^a^		
		2	12.83 ^b^		6.80 ^b^		21.66 ^b^		39.91 ^ab^		28.38 ^c^		25.82 ^ab^		39.16 ^a^		
		3	24.77 ^b^		8.61 ^b^		23.17 ^a^		36.32 ^b^		39.19 ^b^		21.65 ^b^		19.15 ^b^		
Miss		1	58.22 ^a^		38.62 ^a^		24.18 ^a^		55.22 ^b^		68.21 ^a^		25.22 ^b^		58.10 ^a^		
		2	18.27 ^b^		21.82 ^b^		20.56 ^b^		38.99 ^c^		46.49 ^b^		Trace ^c^		4.83 ^c^		
		3	18.89 ^b^		13.10 ^b^		22.13 ^ab^		84.45 ^a^		33.58 ^c^		57.78 ^a^		39.51 ^b^		
Raurica		1	13.01 ^ab^		17.61 ^b^		21.90 ^ab^		29.84 ^b^		58.53 ^a^		62.02 ^a^		43.18 ^a^		
		2	18.16 ^a^		30.55 ^a^		22.13 ^a^		52.60 ^a^		33.06 ^b^		16.98 ^b^		21.76 ^b^		
		3	11.43 ^b^		21.76 ^b^		21.04 ^ab^		12.82 ^c^		29.58 ^b^		37.39 ^b^		23.79 ^b^		

Results with different letters are significantly different (*p* < 0.05); ** *p* < 0.01.

### 2.3. Color Variables in Different Cultivars of Strawberry Fruit

Analyzed color variables in different strawberry cultivars for first and second research year are presented in Table 3 and Table 4. The *a* value in all the cultivars expect ‘Raurica’ was lower in the first research year. There are also significant differences in *a* value between first, second and third harvest period in all analyzed cultivars. The highest *b* value was observed in the ‘Arosa’ cultivar in the second year, while the lowest value was determined in same year for the ‘Miss’ cultivar. Significant differences were noted in *L*, chroma and *h°* between cultivars, year of research and harvest period. ‘Arosa’ cultivar showed lower hue angle and *L* in both years, a synonym of redder and darker colour, while color intensity, *c* was the highest in the first year and also very high in the second year. ‘Raurica’ showed higher *L* during both years, which is in accordance with the observation that they are less red color than the other analyzed strawberry cultivars. Significant differences in *L* and hue *h°* values between harvest times and strawberry pulp color were observed in both years (*p* < 0.01). *L* values in second and third harvest period in both research years are higher. Opposite to that, chroma values were always higher in the first harvest period. Also in second research year, *L* values were lower in first two harvest periods and higher in third harvest period compare with first research year. Cultivar ‘Marmolada’ showed lower values for hue angle and greater values for *a/b* (high color) than the other cultivars, indicating higher coloration. ‘Arosa’ and ‘Elsanta’ showed intermediate *a/b* values, while lower values were recorded for ‘Miss’ followed by ‘Raurica’, the less colored cultivar. During the first year, second harvest produced minimum *a/b* values and minimum values for Hue angle, while during the second year at the same harvest periods those values were maximum for *a/b* values and also very high for Hue angle.

**Table 3:** Effects of cultivar and harvest time on strawberry pulp colour variables during first research year. Values are means ± SD.

			a	b	a/b Ratio	C	L	h°
			**	**	**	**	**	**
**Harvest time**	**First**	‘Arosa’	19.9 ^bc^ ± 0.11	13.3 ^c^ ± 0.58	1.50 ^c^ ± 0.072	27.0 ^a^ ± 0.27	20.3 ^i^ ± 0.41	39.4 ^a^ ± 0.87
		‘Elsanta’	20.8 ^a^ ± 0.49	12.4 ^f^ ± 0.55	1.68 ^ab^ ± 0.086	27.6 ^a^ ± 0.67	21.0 ^h^ ± 0.15	39.7 ^a^ ± 0.39
		‘Marmolada’	19.5 ^bcd^ ± 0.34	13.3 ^c^ ± 0.30	1.46 ^cd^ ± 0.057	23.3 ^d^ ± 0.48	23.7 ^fg^ ± 0.5	32.8 ^d^ ± 0.83
		‘Miss’	19.3 ^de^ ± 0.25	14.6 ^ab^ ± 0.43	1.32 ^efg^ ± 0.050	24.6 ^bc^ ± 0.42	28.4 ^ab^ ± 0.2	39.9 ^a^ ± 0.26
		‘Raurica’	19.2 ^de^ ± 0.18	14.5 ^ab^ ± 0.50	1.32 ^efg^ ± 0.059	24.0 ^c^ ± 0.15	26.9 ^c^ ± 0.23	39.6 ^a^ ± 0.46
	**Second**	‘Arosa’	16.8 ^hi^ ± 0.21	14.2 ^b^ ± 0.33	1.19 ^h^ ± 0.017	20.9 ^e^ ± 0.22	25.9 ^d^ ± 0.34	38.2 ^b^ ± 0.42
		‘Elsanta’	19.4 ^cd^ ± 0.22	13.2 ^cd^ ± 0.32	1.47 ^cd^ ± 0.052	22.9 ^d^ ± 0.28	24.1 ^ef^ ± 0.0	31.8 ^e^ ± 0.36
		‘Marmolada’	17.5 ^g^ ± 0.40	12.6 ^def^ ± 0.25	1.39 ^de^ ± 0.015	20.9 ^e^ ± 0.27	24.4 ^e^ ± 0.47	28.3 ^f^ ± 0.80
		‘Miss’	16.5 ^i^ ± 0.17	13.4 ^c^ ± 0.37	1.24 ^gh^ ± 0.038	19.8 ^f^ ± 0.37	24.4 ^e^ ± 0.27	34.2 ^c^ ± 0.24
		‘Raurica’	18.7 ^ef^ ± 0.42	15.0 ^a^ ± 0.28	1.25 ^gh^ ± 0.050	24.4 ^bc^ ± 0.26	28.7 ^a^ ± 0.21	39.6 ^a^ ± 0.15
	**Third**	‘Arosa’	20.1 ^b^ ± 0.69	11.6 ^g^ ± 0.38	1.73 ^a^ ± 0.094	27.6 ^a^ ± 0.57	21.3 ^h^ ± 0.16	39.1 ^a^ ± 0.94
		‘Elsanta’	18.2 ^f^ ± 0.24	14.5 ^ab^ ± 0.49	1.26 ^gh^ ± 0.058	24.2 ^bc^ ± 0.29	28.0 ^b^ ± 0.08	39.2 ^a^ ± 0.24
		‘Marmolada’	21.2 ^a^ ± 0.41	13.2 ^cde^ ± 0.33	1.61 ^b^ ± 0.017	27.6 ^a^ ± 0.51	21.3 ^h^ ± 0.15	39.9 ^a^ ± 0.20
		‘Miss’	17.2 ^gh^ ± 0.24	12.5 ^ef^ ± 0.48	1.37 ^ef^ ± 0.067	20.5 ^e^ ± 0.32	23.5 ^g^ ± 0.27	32.6 ^de^ ± 0.17
		‘Raurica’	19.4 ^cd^ ± 0.37	15.1 ^a^ ± 0.14	1.29 ^fg^ ± 0.029	24.8 ^b^ ± 0.15	28.5 ^a^ ± 0.12	39.7 ^a^ ± 0.52

a = red-green values; b = blue-yellow values; C = chroma; L = lightness; h° = angle; different letters indicate significant differences between means at *p* ≤ 0.05. * *p* < 0.05, ** *p* < 0.01, NS not significant.

**Table 4:** Effects of cultivar and harvest time on strawberry pulp colour variables during second research year. Values are means ± SD.

			a	b	a/b Ratio	C	L	h°
			**	**	**	**	**	**
**Harvest time**	**First**	‘Arosa’	36.0 ^a^ ± 0.53	24.1 ^a^ ± 0.39	1.49 ^bcd^ ± 0.0	43.1 ^a^ ± 0.21	14.6 ^f^ ± 0.54	33.5 ^ef^ ± 1.04
		‘Elsanta’	21.1 ^d^ ± 0.70	17.9 ^c^ ± 0.16	1.18 ^g^ ± 0.04	27.4 ^d^ ± 0.72	20.8 ^d^ ± 0.52	39.8 ^ab^ ± 0.71
		‘Marmolada’	35.0 ^a^ ± 1.58	22.0 ^b^ ± 0.31	1.59 ^b^ ± 0.05	41.1 ^b^ ± 1.53	13.8 ^f^ ± 1.11	31.5 ^gh^ ± 0.94
		‘Miss’	19.6 ^e^ ± 0.14	12.9 ^fg^ ± 0.52	1.52 ^bcd^ ± 0.00	23.4 ^f^ ± 0.15	23.9 ^c^ ± 0.32	32.8 ^efg^ ± 0.32
		‘Raurica’	19.4 ^e^ ± 0.18	12.6 ^gh^ ± 0.41	1.54 ^bc^ ± 0.04	22.9 ^f^ ± 0.26	24.1 ^c^ ± 0.24	32.1 ^fgh^ ± 1.41
	**Second**	‘Arosa’	16.5 ^i^ ± 0.27	12.4 ^ghi^ ± 0.41	1.32 ^f^ ± 0.05	21.4 ^g^ ± 0.61	24.4 ^c^ ± 0.51	30.2 ^h^ ± 2.53
		‘Elsanta’	32.8 ^b^ ± 0.62	12.1 ^hi^ ± 0.42	2.70 ^a^ ± 0.11	40.7 ^b^ ± 0.82	20.9 ^d^ ± 0.82	36.4 ^c^ ± 0.62
		‘Marmolada’	17.7 ^gh^ ± 0.61	12.4 ^ghi^ ± 0.44	1.43 ^def^ ± 0.09	20.8 ^gh^ ± 0.53	24.5 ^c^ ± 0.44	31.9 ^fgh^ ± 0.81
		‘Miss’	30.2 ^c^ ± 1.53	11.3 ^i^ ± 0.22	2.67 ^a^ ± 0.12	35.4 ^c^ ± 2.31	19.0 ^e^ ± 1.35	30.9 ^gh^ ± 1.87
		‘Raurica’	16.7 ^hi^ ± 0.34	13.8 ^de^ ± 0.30	1.21 ^g^ ± 0.05	21.4 ^g^ ± 0.59	26.1 ^b^ ± 0.43	39.9 ^b^ ± 0.86
	**Third**	‘Arosa’	17.1 ^ghi^ ± 0.01	11.8 ^hi^ ± 0.50	1.45 ^cde^ ± 0.06	20.3 ^gh^ ± 0.34	23.6 ^c^ ± 0.21	32.5 ^efg^ ± 0.33
		‘Elsanta’	16.1 ^i^ ± 0.22	13.6 ^def^ ± 0.47	1.19 ^g^ ± 0.03	19.9 ^h^ ± 0.52	26.0 ^b^ ± 0.83	35.9 ^cd^ ± 1.12
		‘Marmolada’	18.2 ^fg^ ± 0.25	13.4 ^ef^ ± 0.42	1.36 ^ef^ ± 0.06	24.2 ^ef^ ± 0.64	25.9 ^b^ ± 0.34	41.1 ^a^ ± 1.14
		‘Miss’	16.4 ^i^ ± 0.29	12.3 ^ghi^ ± 0.50	1.33 ^f^ ± 0.06	19.8 ^h^ ± 0.35	24.5 ^c^ ± 0.35	4.1 ^de^ ± 0.17
		‘Raurica’	19.2 ^ef^ ± 0.24	14.1 ^d^ ± 0.39	1.36 ^ef^ ± 0.03	24.9 ^e^ ± 0.12	28.5 ^a^ ± 0.01	39.3 ^ab^ ± 0.85

a = red-green values; b = blue-yellow values; C = chroma; L= lightness; h° = angle; different letters indicate significant differences between means at *p* ≤ 0.05, * *p* < 0.05, ** *p* < 0.01, NS not significant.

### 2.4. Correlation Coefficients between Cromacity Parameters and Individual Anthocyanin (Cy 3-gluc, Pg 3-gluc and Pg 3-rut)

A high correlation coefficient (*p* < 0.01) was observed between Cy 3- gluc and *a* (Table 5) in first research year with values ranging from −0.84 (‘Elsanta’) to 0.96 (‘Miss’); while in second research year (Table 5), except for cultivar ‘Elsanta’, no other cultivars show significant correlation between these values. High correlation coefficients between cyanidin 3-glucoside and *b* (0.72) for ‘Miss’ in the first research year and −0.88 for ‘Raurica’ in thesecond research year. A significant correlation was determined for cultivar ‘Elsanta’ (0.62) in the second research year. A high significant correlation was determined between *a/b* ratio and cyanidin 3-glucoside. In the first research it was most evident in cultivars ‘Elsanta’ (−0.79), ‘Raurica’ (0.71) and ‘Marmolada’ (0.85). In the second research year a significant correlation was observed for cultivars ‘Elsanta’ (−0.62), ‘Marmolada’ (−0.53) and ‘Raurica’ (0.85). In the first research year, high correlations were determined between chroma and cyanidin 3-glucoside, particularly in cultivars ‘Marmolada’ (0.88), ‘Miss’ (0.99) and ‘Elsanta’ (−0.57). In the second research year there were no significant correlations between cyanidin 3-glucoside and chroma value. High significant correlations were also determined between cyanidin 3-glucoside and the parameter *L*, in cultivars ‘Marmolada’ (−0.86), ‘Elsanta’ (0.88) and ‘Miss’ (0.92) in the first research year; while in the second research year, significant correlations were observed in cultivars ‘Raurica’ (−0.75) and ‘Marmolada’ (0.58). High correlations were determined between hue angle and cyanidin 3-glucoside, particularly in cultivars ‘Marmolada’ (0.84) and ‘Miss’ (0.91), in the first research year and cultivars ‘Raurica’ (−0.86) and ‘Marmolada’ (0.88) in the second research year.

**Table 5:** Correlation coefficients (r) between chromaticity parameters *a*, *b*, *a/b* ratio, *C*, *L* and *h*° and cyanidin-3-glucoside in five strawberry cultivars in both research years.

First Research Year						
Strawberry Cultivars	a	b	a/b Ratio	C	L	h°
‘Arosa’	−0.00 NS	−0.12 NS	0.08 NS	−0.07 NS	−0.08 NS	0.35 NS
‘Elsanta’	−0.84 **	0.71 **	−0.79 **	−0.57 *	0.88 **	0.02 NS
‘Marmolada’	0.76 **	0.20 NS	0.85 **	0.88 **	−0.86 **	0.84 **
‘Miss’	0.96 **	0.72 **	0.27 NS	0.99 **	0.92 **	0.91 **
‘Raurica’	0.83 **	−0.39 NS	0.71 **	0.04 NS	−0.40 NS	0.08 NS
‘Arosa’	−0.25 NS	−0.30 NS	0.27 NS	−0.31 NS	0.22 NS	0.34 NS
‘Elsanta’	−0.53 *	0.62 *	−0.62 *	−0.50 NS	0.11 NS	0.49 NS
‘Marmolada’	−0.49 NS	−0.46 NS	−0.53 *	−0.39 NS	0.58 *	0.88 **
‘Miss’	0.02 NS	0.49 NS	−0.08 NS	0.02 NS	0.10 NS	0.02 NS
‘Raurica’	0.50 NS	−0.88 **	0.85 **	−0.04 NS	−0.75 **	−0.86 **

Correlation coefficients between each chromaticity value and cyaniding 3-glucoside with statistically significant; differences of the estimated correlations presented as * *p* < 0.05, ** *p* < 0.01, NS not significant.

Additionally, the correlation coefficient between chromaticity parameters and the second major strawberry anthocyanin, pelargonidin 3-glucoside, was determined in both research years (Table 6). In the first research year significant correlations were observed between pelargonidin 3-glucoside and *a* (‘Marmolada’, 0.92 and ‘Miss’ −0.89); *b* (‘Marmolada’, 0.80; ‘Miss’, −0.89 and ‘Raurica’, −0.54); *a/b* ratio (‘Marmolada’, 0.77); *h°* (‘Marmolada’, 0.86; ‘Miss’, −0.99 and ‘Elsanta’ 0.58) and *L* (‘Marmolada’, 0.84; ‘Miss’, −0.93 and ‘Raurica’, −0.88). However, in the second research year results were slightly different. Significant correlations were observed between pelargonidin 3-glucoside and *a* (‘Raurica’, 0.71 and ‘Arosa’, −0.92), *b* (‘Marmolada’, 0.53; ‘Miss’, −0.77 and ‘Arosa’, −0.94), *h°* (‘Marmolada’, 0.51), *L* (‘Arosa’, 0.90 and ‘Raurica’, 0.66), while a correlation between pelargonidin 3-glucoside and *a/b* ratio wasn’t noted in the analyzed cultivars.

**Table 6:** Correlation coefficients (r) between chromaticity parameters *a*, *b*, *a/b* ratio, *C*, *L* and *h*° and pelargonidin 3-glucoside in five strawberry cultivars in both research years.

First Research Year						
Strawberry Cultivars	a	b	a/b Ratio	C	L	h°
‘Arosa’	−0.21 NS	0.36 NS	0.34 NS	0.21 NS	−0.02 NS	−0.04 NS
‘Elsanta’	−0.36 NS	0.50 NS	−0.40NS	0.13 NS	0.39 NS	0.58 *
‘Marmolada’	0.92 **	0.80 **	0.77 **	0.84 **	−0.74 **	0.86 **
‘Miss’	−0.89 **	−0.89 **	0.04 NS	−0.93 **	−0.98 **	−0.99 **
‘Raurica’	−0.32 NS	−0.54 *	0.19 NS	−0.88 **	−0.70 **	−0.09 NS
**Second Research Year**						
‘Arosa’	−0.92 **	−0.94 **	−0.34 NS	−0.94**	0.90 **	−0.32 NS
‘Elsanta’	−0.04 NS	0.23 NS	−0.09 NS	−0.03 NS	−0.14 NS	0.15 NS
‘Marmolada’	0.46 NS	0.53 *	0.15 NS	0.57*	−0.34 NS	0.51 *
‘Miss’	0.40 NS	−0.77 **	0.49 NS	0.41 NS	−0.50 NS	−0.11 NS
‘Raurica’	0.71 **	0.31 NS	0.31 NS	0.97 **	0.66 **	0.20 NS

Correlation coefficients between each chromaticity value and pelargonidin 3-glucoside with statistically; significant differences of the estimated correlations presented as * *p* < 0.05, ** *p* < 0.01, NS not significant.

**Table 7:** Correlation coefficients (r) between chromaticity parameters *a*, *b*, *a/b* ratio, *C*, *L* and *h*° and pelargonidin-3-rutinoside in five strawberry cultivars in both research years.

First Research Year						
Strawberry Cultivars	a	b	a/b Ratio	C	L	h°
‘Arosa’	0.59 *	−0.94 **	0.85 **	0.63 *	−0.44 NS	0.15 NS
‘Elsanta’	−0.96 **	0.97 **	−0.99 **	−0.66 **	0.93 **	−0.09 NS
‘Marmolada’	−0.50 NS	0.13 NS	−0.67 **	−0.67 **	0.78 **	−0.66 **
‘Miss’	0.77 **	0.93 **	−0.25 NS	0.77 **	0.92 **	0.94 **
‘Raurica’	−0.10 NS	−0.68 **	0.42 NS	−0.85 **	−0.90 **	0.02 NS
**Second research year**						
‘Arosa’	0.90 **	0.88 **	0.71 **	0.89 **	−0.90 **	0.52 *
‘Elsanta’	−0.31 NS	−0.61 *	−0.06 NS	−0.36 NS	0.81 **	−0.70 **
‘Marmolada’	0.98 **	0.96 **	0.87 **	0.95 **	−0.98 **	−0.59 *
‘Miss’	−0.92 **	0.82 **	−0.94 **	−0.93 **	0.93 **	0.50 NS
‘Raurica’	−0.38 **	−0.62 *	0.10 NS	−0.83 **	−0.88 **	−0.54 *

Correlation coefficients (*r*) between each chromaticity value and pelargonidin-3-rutinoside with statistically significant; differences of the estimated correlations presented as * *P* < 0.05, ** *P* < 0.01, NS not significant.

A high correlation coefficient was observed between pelargonidin 3-rutinoside and *a* (Table 7) in the first research year with values of −0.96 (‘Elsanta’), 0.77 (‘Miss’) and 0.59 (‘Arosa’), while in the second research year, except for cultivkar ‘Elsanta’, a high statistically significance of the estimated correlations were observed in all cultivars ranging from −0.92 (‘Miss’) to 0.98 (‘Marmolada’). In the first research year, significant correlations were observed between pelargonidin 3-rutinoside and *b* (‘Arosa’, −0.94; ‘Raurica’, −0.68; ‘Miss’, −0.68 and ‘Elsanta’, 0.97), *a/b* ratio (‘Elsanta’, −0.99; ‘Marmolada’, −0.67 and ‘Arosa’, 0.85), chroma (‘Raurica’, −0.85; ‘Marmolada’, −0.67; ‘Elsanta’, −0.66; ‘Miss’, 0.77 and ‘Arosa’, −0.63), *h°* (‘Raurica’, −0.90; ‘Marmolada’, 0.78; ‘Miss’, 0.92 and ‘Elsanta’, 0.93), and *L* (‘Marmolada’, −0.99; ‘Miss’, 0.94). In the second research year, significant correlations were observed between pelargonidine 3-rutinoside and *b* (‘Miss’, 0.82; ‘Arosa’, 0.88; ‘Marmolada’, 0.96; ‘Raurica’, −0.62 and ‘Elsanta’, −0.61), *a/b* ratio (‘Miss’, −0.94; ‘Arosa’, 0.71 and ‘Marmolada’, 0.87), chroma (‘Miss’, −0.93 ‘Raurica’, −0.83; ‘Arosa’, 0.89 and ‘Marmolada’, 0.95), *L* (ranged from 0.98 ‘Marmolada’ to 0.93 ‘Miss’), *h*° (‘Elsanta’, −0.51; ‘Marmolada’, −0.59; ‘Raurica’, −0.54 and ‘Arosa’, 0.52).

## 3. Discussion

### 3.1. The Individual Anthocyanin Composition of Selected Strawberry Varieties

HPLC determination of individual anthocyanins in this research suggests the presence of three major anthocyanins: Pg 3-gluc, Cy 3-gluc and Pg 3-rut (3.1.). Similar results by individual anthocyanin content were reported by other authors [17,18]. According to the literature citations Pg 3-malonylglucoside was the second most abundant anthocyanin in the strawberry samples, varying from 0% to 33.5%. In general, the content of individual anthocyanins is influenced significantly by strawberry cultivar, harvest period and climatic conditions, as also indicated in a number of literature citations [3,6,19]. The proportions found among Pg 3-gluc, Cy 3-gluc and Pg 3-rut were consistent for analyzed samples of the same variety, suggesting a characteristic anthocyanin distribution. A significant difference in the individual anthocyanins content between the studied strawberry varieties in different harvest stage in both research years is directly correlated to the edaphic and climatic factors [20]. Comparisons of the anthocyanin composition of analyzed strawberry cultivars during both research years can be used to determine the highly significant differences. The main reasons for the large differences between the two years of research is because of climatic conditions, the values of temperature and precipitation (Table 8 and Table 9) were significantly different. Also, day-length must not be neglected and together with temperature, both influenced by latitude, are the most important factors influencing on basic nutritional composition of strawberry fruits [21].

### 3.2. Phenolic Compounds Determined in Selected Strawberry Varieties

Strawberry fruits are a rich source of many bioactive phytochemicals for human consumption. Phenol compounds that are found in strawberries include anthocyanins, flavonols, catechins and proanthocyanidins [18,22]. The determined phenolic acids (caffeic acid, chlorogenic acid and ellagic acid) content in the strawberry varieties analyzed in this research are in agreement with the literature data of other authors [18,22]. High contents of ellagic acid in the five analyzed strawberry cultivars are a very important characteristic of strawberry fruit because ellagic acid is a natural antimutagenic and anticancer compound [15,23]. Concentrations of analyzed phenol compounds (phenolic acids and falvonoids) differed with strawberry cultivar, but the changes during ripening were ambiguous. Highly significant statistical differences were determined for phenol composition in analyzed strawberry cultivars (*p* < 0.01), respectively. Strawberry genotype strongly affects a wide range of phenol values from total flavonoids to phenolic acids. Obtained results are in agreement with the literature data [18,24].

### 3.3. Color Variables in Different Cultivars of Strawberry Fruit

The pulp color was dependent on harvest period with high statistical differences. Fruit color is also a cultivar characteristic, so the differences among them are to be expected. Effects of cultivar and harvest period on strawberry pulp color parameters for the first year and second year of research shows considerably significant differences (*p* < 0.001). The results show a positive association between cultivar and harvest period on strawberry pulp color, with each of the color variables (*a*, *b*, *a/b* ratio, *C*, *L* and *h°* values). The decrease in chroma means an increase in the tonality of the fruit color. The decrease of chroma values could have been caused by an increase in total anthocyanin content and a decrease in chlorophyll composition, and according to [3] and [19], by development of dark, pigmented compounds, which tend to mask color. According to the harvest time and pulp color variables, data presented above (Section 2.3) shows that the strawberry colors are satisfying even at early stages of commercial harvest. Fruits from all analyzed cultivars show high values of color variables at all three harvest periods. However, the changes in colorimetric parameters varied depending on climate conditions and the anthocyanin composition in the different strawberry cultivars.

### 3.4. Correlation Between Cromacity Parameters and Individual Anthocyanins (Cy 3-gluc, Pg 3-gluc and Pg 3-rut)

The high correlation coefficient between cyanidin 3-glucoside content and cromacity parameter *a* determined for cultivars ‘Elsanta’ and ‘Miss’ suggesting that the analyzed strawberry fruits with high determined content of Cy 3-gluc present a characteristic intensive red color which shows a strong connection between anthocyanin content and the development of fruit color. Strawberry fruits of all analyzed cultivars contained only small amounts of pelargonidine 3-rutinoside. Despite that fact, the pelargonidine 3-rutinoside content in strawberry cultivars correlates with all analyzed color measurements more than the other two anthocyanins and can be directly linked to the red color in most of the studied cultivars. Obtained results of correlation coefficients between pelargonidine 3-rutinoside and the parameter *a* suggest that Pg 3-rut is responsible for the expression of red color in the fruits of all analyzed cultivars. The correlation coefficient between color variables (*b*, *L*, *C*, hue angle) and the second major anthocyanin, pelargonidin 3-rutinoside, was calculated too but also could not be generalized to all cultivars [25,26]. In all analyzed strawberry cultivars the correlation coefficients between color variables and Pg 3-rut were high, which assumes a strong connection between the full red characteristic strawberry color and satisfactory content of Pg 3-rut. Determination of color variables and correlation between characteristic cultivar color and individual anthocyanin profile of strawberry fruits has always been variable, primarily due to the effect of many other parameters that are involved in color stability of fresh fruits, such as pH, temperature, light, oxygen, enzymatic and non-enzymatic reactions and l-ascorbic acid (l-AA) [27]. Color stability is influenced by process of anthocyanin condensation [28] and copigmentation (interaction of anthocyanins with polyphenols) [29,30].

## 4. Experimental Section

### 4.1. Fruit Material

Cold stored (frigo) strawberry plants of cultivars ‘Arosa’, ‘Elsanta’, ‘Marmolada’, ‘Miss’ and ‘Raurica’ were planted in black plastic foil. Fertilizers and water were provided by fertirrigation. The strawberries were planted at a planting density of 40.000 plants/ha in an open field on alluvium soil in the Zagreb area. The above mentioned strawberry cultivars for the purpose of this research were selected for the main reason of examining the possibilities for extending the consumption season of strawberry fruits. Namely, earlier maturity strawberry varieties (‘Elsanta’, ‘Miss’) until later strawberry varieties (‘Raurica’) were studied. The experiment was designed as a random block design with three blocks. Fruits were harvested during the end of May and through June in two research years (Table 8) with average of 29.9 mm precipitation in the first year and 85.8 mm precipitation in the second research year (Table 9); mean air temperature of 19.9 °C in the irst and 20.5 °C in the second research year (Table 10). Also strawberry fruits were harvested when the fruits were at full maturity which is fundamentally determined by the appearance of their characteristic red coloration. Strawberry fruits were stored at −20 °C until intended chemical analysis. The following quality parameters of harvested fruits were determined: individual anthocyanins, fruit color variables and content of phenolic compounds.

**Table 8:** Harvest dates of strawberry cultivars in two years of research.

Cultivar	Year of Research					
	1st Year Harvest Date			2nd Year Harvest Date		
	1.	2.	3.	1.	2.	3.
‘Arosa’	02 Junuary	10 Junuary	17 Junuary	29 May	06 Junuary	12 Junuary
‘Elsanta’	02 Junuary	10 Junuary	17 Junuary	24 May	31 May	07 Junuary
‘Marmolada’	02 Junuary	10 Junuary	17 Junuary	29 May	06 Junuary	12 Junuary
‘Miss’	02 Junuary	10 Junuary	17 Junuary	24 May	31 May	07 Junuary
‘Raurica’	02 Junuary	10 Junuary	17 Junuary	01 Junuary	07 Junuary	12 Junuary

**Table 9:** Rainfall (mm) during the two research years.

Year	Decade	January	February	March	April	May	June
First	I	2.4	11.0	13.4	37.5	40.5	24.4
	II	7.5	26.7	9.7	21.3	42.4	3.4
	III	17.8	36.2	33.1	12.0	0.0	2.1
	**Amount**	**27.7**	**73.9**	**56.2**	**70.8**	**82.9**	**29.9**
Second	I	53.1	0.6	27.6	52.5	23.5	54.5
	II	0.6	7.2	24.9	37.4	2.6	17.1
	III	0.1	33.4	19.6	40.6	68.4	14.2
	**Amount**	**53.8**	**41.2**	**72.1**	**130.5**	**94.5**	**85.8**

**Table 10:** Mean air temperature during the two years of research (°C).

Year	Decade	January	February	March	April	May	June
First	I	2.8	−6.6	−3.8	10.0	14.1	15.7
	II	−1.7	1.2	7.5	11.5	14.8	20.1
	III	−3.5	−1.0	11.3	13.5	21.2	23.9
	Mean value	−0.7	−2.2	5.0	11.4	16.7	19.9
Second	I	0.4	−2.3	1.4	10.6	13.2	13.4
	II	−1.5	3.9	2.6	12.2	17.8	22.3
	III	−3.8	2.1	10.4	14.2	18.0	25.8
	Mean value	−1.7	1.2	5.0	12.3	15.8	20.5

### 4.2. Chemicals

Anthocyanin-glucoside (cyanidin-3-*O*-glucoside, pelargonidin 3-glucoside and pelargonidin 3-rutinoside) and phenol standards (caffeic acid, chlorogenic acid, ellagic acid, epicatehin, catehin, procyanidin B2 and rutin) were purchased from Extrasynthèse (Genay, France), HPLC grade methanol and formic acid were obtained from Merck (Darmstadt, Germany).

### 4.3. Determination of Fruit Color

The color values were expressed as CIE *Lab* (CIELab) values. The color is defined three dimensionally using the *L*, *a*, *b* notation. The *L* axis represents lightness of the color (the lower the value, the darker the color). *L* is orthogonal to a chromaticity plane defined by two perpendicular axes, *a* and *b*. The *a* axis represents the balance between red (positive values) and green (negative values) and the *b* axis the balance between yellow (positive values) and blue (negative values). These coordinates give access to new indices, the hue angle (*h* = arctan *b/a*), which represents the basic color. Internal flesh (pulp) color, (each side of a longitudinally sliced fruit) was measured using a ColorTec colorimeter.

### 4.4. HPLC Determination of Individual Anthocyanin and Phenol Compounds in Strawberry Cultivars

For HPLC analysis fruits were mashed in a laboratory homogeniser (Mixy, Zepter International, Zürich, Switzerland) in order to obtain natural fruit mash for anthocyanin extraction. Phenolic compounds were recovered from the strawberry tissues by extraction with aqueous methanol in a nitrogen atmosphere. The mashed fruit material (5 g) after addition of ascorbic acid (0.1 g, to inactivate polyphenol oxidases and prevent phenolic degradation due to browning) was homogenized with 20 mL of extraction solution (80% aqueous methanol). This mixture was homogenized for 10 min and filtered through a Whatman filter paper using a Büchner funnel. Filtrate was transferred to 25 mL volumetric flask and filled to its mark with 80% aqueous methanol. The sample solutions were stored at −20 °C in inert atmosphere until analysis. Part of prepared samples was filtered through 0.45 μm syringe filter (VariSep PTFE, 0.45 μm, 25 mm-Varian) prior to injection into the HPLC. The composition of solvents and used gradient elution conditions were as described previously by Tomás-Barberán *et al.* [31] with some modification. Formic acid (2.5%) was added to water and methanol and following mobile phases were set up: 95% water + 5% methanol (A); 88% water + 12% MeOH (B) and 20% water + 80% MeOH (C). The following gradient elution was used for the separation of individual anthocyanins and phenol compounds: 100% A was held isocratic until 5 min, at 15 min gradient elution was reach to 100% B, held isocratic for 3 more minutes. From 18 to 35 min a linear gradient was used to reach 75% B and 25% C, and then 50% B and 50% C at 50 min, and 100% C at 52 min, then maintained isocratic until 57 min. The column was then washed with 100% A at 60 min. Operating conditions were as follows: flow rate 1.0 mL min^−1^, column temperature 20 °C, injection volume 20 μL of the standards and sample extracts. The measurements were performed on a UV/VIS-photodiode array detector with the highest detection sensitivity at 510 nm. The analytical HPLC system employed consisted of a Varian LC system (Santa Clara, CA, USA) equipped with a ProStar 230 solvent delivery module, ProStar 330 UV-Vis Detector, and ProStar 330 Photodiode Array Detector (PDA) Detector. Anthocyanin separation was done in a Nucleosil C-18 5 µm (250 × 4.6 mm I.D.) column equipped with a Nucleosil C-18 guard column, 5 µm (10 × 4.6 mm I.D.).

### 4.5. Statistical Analysis

According to a randomized block design of the field experiment (with three replicates), ANOVA and Duncan’s multiple range tests were performed to determine the significance of differences within the examined factors (harvest time and cultivar) and between the combination of factors (where interaction was proven significant), using the commercial software 9.3 (SAS 2010) [32]. Values are presented as the mean ± SD of three replications. *p*-values lower than 0.05, either from ANOVA or Duncan’s multiple range test were considered statistically significant. Regression analysis was calculated for cyanidin-3-glucoside, pelargonidine-3-glucoside, and pelargonidine-3-rutinoside and each of the color variables estimated with a portable colorimeter.

## 5. Conclusions

Strawberry fruits are a good source of antioxidative compounds. The results of this study confirm that climatic conditions during the ripening of fruits significantly affect the concentration of phenolic compounds and individual anthocyanin content. Statistical analysis of differences between the cultivars showed that cultivar has the main impact on the concentration of anthocyanin and the pulp color. The results show a positive association between cultivar and harvest time on strawberry pulp color, with each of the color parameters, *a*, *b*, *a/b* ratio, *C*, *L* and *h°* values. Color of strawberry fruits is an important quality attribute. Measuring color parameters can be used to monitor pigment evolution and anthocyanin content and can provide an objective judgment of food quality. Climatic conditions, agricultural techniques and type of strawberry varieties greatly affect the presence of the individual phenolic compound anthocyanins characteristic of strawberry fruit. Therefore, for strawberry fruits intended for human consumption, it seems important to have a simple and non-destructive technique for anthocyanin content determination, and in this way easily and quickly assess and monitor strawberry fruit quality on a large number of cultivars.
